# Physiologically Based Pharmacokinetic Models for Infliximab, Ipilimumab, and Nivolumab Developed with GastroPlus to Predict Hepatic Concentrations

**DOI:** 10.3390/pharmaceutics17030372

**Published:** 2025-03-14

**Authors:** Celeste Vallejo, Cameron Meaney, Lara Clemens, Kyunghee Yang, Viera Lukacova, Haiying Zhou

**Affiliations:** Simulations Plus, Research Triangle Park, Durham, NC 27709, USA; cameron.meaney@simulations-plus.com (C.M.); lara.clemens@simulations-plus.com (L.C.); kyunghee.yang@simulations-plus.com (K.Y.); viera.lukacova@simulations-plus.com (V.L.)

**Keywords:** physiologically based pharmacokinetic model, infliximab, ipilimumab, nivolumab, large molecule, hepatic concentration

## Abstract

**Background/Objectives:** Infliximab, ipilimumab, and nivolumab are three monoclonal antibodies that have been associated with hepatotoxicity. Three separate physiologically based pharmacokinetic (PBPK) models were developed in GastroPlus^®^ to simulate plasma and liver concentrations in patient populations after administration of either infliximab, ipilimumab, or nivolumab. **Methods:** The models include distribution and clearance mechanisms specific to large molecules, FcRn binding dynamics, and target-mediated drug disposition (TNF-α for infliximab, CTLA-4 for ipilimumab, and PD-1 for nivolumab). **Results:** The PBPK model for each large molecule was able to reproduce observed plasma concentration data in patient populations, including patients with rheumatoid arthritis and patients with solid tumors. Liver concentrations were predicted to be between 10% and 23% of the plasma concentrations for each of the three drugs, aligning with previously reported results. This lends further validity to the PBPK models and their ability to accurately predict hepatic concentrations in the absence of direct tissue measurements. **Conclusions:** These results can be used to drive liver toxicity predictions using the quantitative systems toxicology model, BIOLOGXsym™, which integrates hepatic interstitial concentrations with in vitro mechanistic toxicity data to predict the extent of liver toxicity for biologics.

## 1. Introduction

Large molecules are becoming more frequently used to treat a wide variety of conditions such as rheumatoid arthritis (RA) and melanoma. Understanding drug exposure in various tissues is critical in evaluating potential efficacy or toxicity. For example, to predict liability for liver toxicity of large molecules, it is important to obtain accurate estimates of the compound’s liver interstitial concentrations. Measuring hepatic drug concentrations directly is challenging, which makes physiologically-based pharmacokinetic (PBPK) modeling an important component of drug development.

PBPK models are a useful tool in predicting tissue concentration after drug administration [1,2,3]. These models rely on both drug properties as well as physiology to assess drug disposition, including tissue distribution. GastroPlus^®^ is a PBPK simulation modeling platform suitable for (among other applications) predicting hepatic concentrations of a drug [4]. The Biologics Module of GastroPlus is specifically designed to capture the processes unique to large molecules, including distribution into the tissue through endocytosis, as well as catabolism within the tissue.

One potential drawback to making predictions using a PBPK model is that there are often a large number of parameters that are used within the model and, if not properly constrained, many different parameter combinations may lead to the same model output. Therefore, it is important to use sufficient data such that the parameters can be constrained as much as possible to increase their interpretability. This could come from the inclusion of plasma concentration time data from multiple dose routes, multiple dose levels, or tissue concentrations. The former two are more likely to be available than the latter due to sampling difficulty. However, indirect validation of tissue concentration may be one way in which to utilize this information without it being directly sampled. Shah et al. [5] developed an empirical model to predict tissue concentrations from plasma concentrations after administration of large molecules across many species using experimental plasma and tissue concentrations. This type of model can be useful in further validating a PBPK model by assessing the quality of its tissue-level predictions.

In the current study, PBPK models for infliximab, ipilimumab, and nivolumab were developed and validated using the GastroPlus Biologics Module across several dose levels. Infliximab, ipilimumab, and nivolumab are three large molecules associated with hepatotoxicity [6,7,8]. Infliximab is a recombinant DNA-derived chimeric IgG monoclonal antibody that contains both murine and human components [7]. It binds to and inhibits tumor necrosis factor alpha (TNF-α) to prevent an inflammatory reaction and is used to treat conditions such as RA, psoriasis, and Crohn’s disease [7]. Ipilimumab is an IgG1 antibody used in the treatment of cancers such as melanoma [8,9]. It is a cytotoxic T-lymphocyte associated protein 4 (CTLA-4) immune checkpoint inhibitor that blocks T-cell inhibitory signals induced by the CTLA-4 pathway [8]. Nivolumab is an IgG4 antibody used in the treatment of various cancers including non-small cell lung cancer, renal cell carcinoma, and melanoma [6]. It binds to programmed cell death protein 1 (PD-1), an inhibitory receptor expressed on the surface of activated immune cells, blocking immune inhibition and increasing the anticancer action of immune cells [6]. Parameterizations for both disposition of the drugs in normal healthy volunteer (NHV) populations as well as patient populations (such as RA) were explored when data were available. For all three compounds only plasma concentration time data were measured. The Shah empirical model [5] was utilized as an additional source of validation. Although this approach has been explored and so is not completely novel [3], it is more common that indirect tissue validation is not used in the weight of evidence for validation in addition to the reproduction of exposure parameters [10,11,12], and, thus, this paper gives further validity to using information that was not directly measured.

The purpose of this paper is to show the development, optimization, and validation of three PBPK models for the large molecules infliximab, ipilimumab, and nivolumab. The models are intended to be used for predicting liver concentrations for use in the quantitative systems toxicology (QST) platform called BIOLOGXsym™ version 0.5 [1] for liver toxicity predictions. Therefore, since liver concentrations were not directly measured, it is important to have assurance regarding the predicted liver concentrations. The liver concentrations for each of the large molecules predicted by their respective PBPK models fell within two-fold of the Shah empirical model predictions, which were developed using clinical data. Validation of the PBPK models against both plasma and liver concentrations provides reasonable support in the quality of the models’ predictions.

## 2. Materials and Methods

### 2.1. PBPK Modeling Software

**Figure 1:**
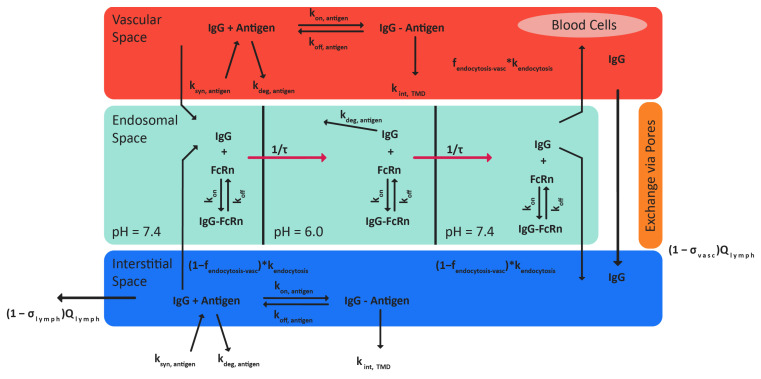
Diagram of tissue subcompartments within the GastroPlus Biologics Module. Distribution processes allow the large molecule to enter the endosomal space (green) from either the vascular (red) or interstitial space (blue), the interstitial space from the vascular space, or out of the tissue from the interstitial space. Clearance mechanisms are present in both the second endosomal subspace as well as in the interstitial or vascular space when binding antigen is incorporated.

The GastroPlus^®^ version 9.9 Biologics Module (Simulations Plus, Research Triangle Park, Durham, NC, USA) [4] was used to build the PBPK models for each of the three monoclonal antibodies. All model equations are included in the Supplementary Materials. The Biologics Module contains distribution and clearance mechanisms specific to large molecules. The tissue subspaces relevant for modeling biologics are the vascular, endosomal (itself divided into three subspaces), and interstitial space (Figure 1). All tissues are connected by both blood and lymph flow. Convective transport distributes the antibody from the tissue’s vascular space into the interstitial space and out of the interstitial space into the lymph. Fluid phase endocytosis distributes the antibody from either the interstitial space or the vascular space into the first endosomal subspace and from the third endosomal subspace back to either the vascular or interstitial space.

Clearance can either occur as a result of unsuccessful binding to the neonatal Fc receptor (FcRn) and subsequent lysosomal degradation or through internalization of the antigen–antibody complex if applicable. The endosomal subspace is divided into three compartments to capture the change in pH with the formation of the endosome that results from uptake of the large molecule. Since binding to the FcRn is pH-dependent, it is important to account for pH differences. The first endosomal subspace represents the initial uptake of the large molecule by endocytosis when the endosome has not been completely formed. At this point, the environment has a pH of 7.4 which is associated with lower binding affinity between the antibody and the FcRn. The second endosomal subspace represents the complete formation of the endosome with a pH of 6 [13], associated with higher binding affinity between the antibody and the FcRn. It is in this compartment that clearance of the antibody can occur (degradation by the lysosome) if the antibody does not successfully bind to the FcRn [14]. The third endosomal subspace represents the recycling of the antibody back to the cell surface. The pH of this compartment is 7.4, allowing the antibody to unbind from the FcRn and be recycled back to the vascular or interstitial space. If the drug binding to the target antigen impacts the drug’s PK, the processes associated with target-mediated drug distribution (TMDD) may be included in the model. Separate dynamics for antigen synthesis and degradation and tissue-specific antigen expression are incorporated, as well as binding dynamics of the antibody and antigen. Antibody bound to the antigen can be cleared through internalization of the complex [15].

Default parameters within the software, such as vascular and lymph reflection coefficients, endosomal uptake and recycling rate, pH-specific binding and unbinding rates to FcRn, and the lysosomal degradation rate, were calibrated using IgG-type antibody data [16]. Default physiological parameters such as lymph flow rate [17,18] and FcRn tissue-level expression [19,20] were taken from the literature. Any other physiological overlap with small molecules, including organ size and blood perfusion rate, used the same values default for small molecules [4]. Endogenous IgG is also included as a physiological parameter within the model. Endogenous IgG competes with the exogenous compound for binding to the FcRn and thus can affect clearance of the exogenous compound. Default parameter values for endogenous IgG were the same as the default for the exogenous IgG compound, as they were calibrated using the same data. The biologics module in GastroPlus 9.9 incorporates distribution and mechanisms related to IgG type antibodies. It is not suitable for smaller proteins.

### 2.2. Infliximab PBPK Model Development

#### 2.2.1. TNF-α Representation in Infliximab PBPK Model

Infliximab’s mechanism of action is binding to and inhibiting TNF-α to prevent inflammatory reactions [7]. TNF-α levels are elevated in patient populations with diseases characterized by increased inflammation, such as RA [21]. Therefore, TNF-α was incorporated into the model as an antigen to which infliximab could bind, introducing TMDD into the model. Infliximab is known to bind to TNF-α both in the plasma and also on the tissue membrane [22]. However, there was insufficient information in the literature to inform TNF-α tissue distribution. Therefore, within the PBPK model, TNF-α was only represented in the plasma. The active form of TNF-α exists as a homotrimer [22]. Infliximab is able to bind to monomer subunits of TNF-α [22]. This would potentially indicate that more than one infliximab molecule could bind to active TNF-α and thus would not preserve a one-to-one binding ratio. There does not appear to be a quantification assay that can explicitly measure the form of TNF-α (trimer vs. monomer). Therefore, it was assumed that TNF-α only exists in its monomer form in the PBPK model and that there is a one-to-one binding ratio between antibody (infliximab) and antigen (TNF-α). Additionally, a separate TNF-α concentration level was used for NHV and RA patients [21]. Parameters related to antigen incorporation into the PBPK model are given in Table 1. Most parameters were found in the literature. No optimization was needed for antigen parameterization in the PBPK model. The previously validated value for TNF-α degradation rate from the QST model [1] is also used in the PBPK model.

#### 2.2.2. PBPK Model Development for Infliximab

**Figure 2:**
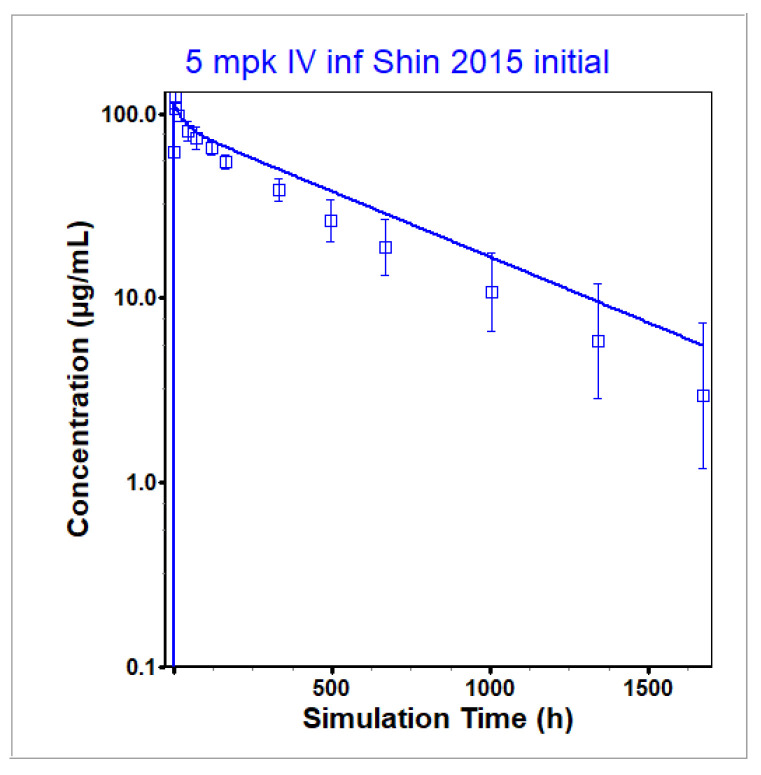
Initial NHV model prediction compared to infliximab data from Shin et al. [28]. Observed data are given by the squares, and the average model prediction is represented by the line. Total and unbound concentrations lie on the same line. The *y*-axis is on a log scale. Clearance is underpredicted using default GastroPlus values in combination with model inputs taken from the literature.

The PBPK model for infliximab utilized the structure for large molecules in GastroPlus described in Section 2.1 with the inclusion of endogenous IgG and TNF-α antigen. The table of model input parameters taken from the literature for infliximab and TNF-α dynamics is given in Table 1. The initial NHV PBPK model, fit to the dataset of Shin et al. [28] using the literature-derived parameter values (Table 1) along with the default GastroPlus parameters for an IgG molecule [4], is given in Figure 2 and shows that clearance is slightly underpredicted. Unbound and total infliximab concentrations lie on top of each other, meaning that unbound concentrations are equal to total infliximab plasma concentrations at this dosing level and antigen expression level. A sensitivity analysis for TNF-α levels shows that within physiological range, unbound and total infliximab concentrations are equal (see Figure S1 in the Supplementary Materials). To match the PK profile in Shin et al. [28], the unbinding rate (Koff) to FcRn in pH 6 was increased from 500 per day to 800 per day. All other parameters were taken to be the default value for IgG molecules provided in GastroPlus. Final model parameters are given in Table 2. All default parameter values were used for the endogenous IgG present in the model. The quality and accuracy of the model predictions were judged by visual comparison of simulated and observed plasma concentration time profiles and by comparison of predicted and observed C_max_ and AUCs, with simulated/observed ratios in the range of 0.8 to 1.25 considered accurate, 0.5 to 2 considered adequate, and ratios <0.5 or >2 (i.e., more than 2-fold error) deemed inadequate.

#### 2.2.3. Accounting for the Development of Anti-Drug Antibodies (ADAs)

NHV individuals that subsequently tested positive for anti-drug antibodies (ADAs) to infliximab had a PK profile that showed higher clearance compared to individuals that were negative for ADAs. Due to the PK differences, the same model would not be applicable to both ADA positive and ADA negative individuals. Therefore, it was important to incorporate the effects of ADA development on the PK after dosing of infliximab. Biologics that target aspects of the immune system have the potential to encourage the development of ADAs. ADA development can put patients at risk for immune response due to the ADA formation, loss of drug targeting, and the formation of highly immunogenic complexes [29]. Further, for drugs such as infliximab, ADAs can cause changes to the PK, including increased clearance [29], which can lead to a reduction in efficacy [30]. Combination therapies with infliximab (such as with methotrexate) have been found to induce less ADA development [31], but do not reduce the prevalence to zero. Due to the lack of data surrounding ADA development in infliximab treatment, a mechanistic model cannot be parameterized. However, a model accounting for the empirical increase in clearance observed in patients that developed ADAs after infliximab treatment can be parameterized. The authors in [29] note that ADAs can bind to the Fc-region of the antibody. This would prevent the antibody from binding to the Fc receptor, protecting it from achieving clearance in the lysosomes. Therefore, in order to reproduce this increased clearance in ADA-positive patients in the infliximab PBPK model, the degradation rate in the lysosomes (Kdeg) was increased from the default 10,000 per day for the default IgG antibody to 12,500 per day. The modified value was optimized against the mean profile from Shin et al. [28], as this data represents the plasma concentration time profile after a single dose of infliximab only in NHV individuals that subsequently tested positive for ADAs.

### 2.3. Ipilimumab PBPK Model Development

#### 2.3.1. CTLA-4 Representation in Ipilimumab PBPK Model

CTLA-4 is expressed on T cells and acts as a checkpoint inhibitor of the immune system [9]. When upregulated, CTLA-4 blocks T-cell signaling, and downregulates the adaptive immune response [9]. Ipilimumab’s mechanism of action is to bind to CTLA-4 on the surface of the T cell (mainly) to prevent the block of T-cell signaling [32]. It is primarily indicated for treating cancers, where amplifying a person’s natural immune response to cancer cells through increased activation may improve patient outcomes. CTLA-4 was incorporated into the ipilimumab PBPK model as an antigen to which it could bind, thereby incorporating TMDD into the model. Since neither T cells nor expression of CTLA-4 on T cells can be explicitly represented in GastroPlus, a simplification was made such that all CTLA-4 was represented as a soluble antigen in the blood compartment. For consistency with interpretation of CTLA-4 form, the cytoplasmic CTLA-4 half-life was used to calculate the CTLA-4 decay rate [33]. The difference in half-life between cytoplasmic and surface bound CTLA-4 is approximately 40 min. CTLA-4 concentration in the blood was one of the parameters that required optimization to match the observed PK given published values for ipilimumab’s binding to CTLA-4. Although it was assumed that all CTLA-4 in the model was soluble, the total concentration of CTLA-4 in the model needed to account for both membrane and soluble CTLA-4 concentrations. CTLA-4 plasma concentrations for healthy and various patient populations that could be found in the literature [34] needed to be augmented to include membrane-bound CTLA-4 concentrations. However, these are difficult to measure as CTLA-4 is not readily detectable at the cell’s surface due to its rapid internalization rate [35,36]. It has been shown that the total amount of CTLA-4 in activated peripheral T cells is comparable to CD28 [36]. Therefore, CD28 concentrations, which are easier to measure since they are primarily located on the cell surface while CTLA-4 is primarily located in intracellular compartments [36], were used as a surrogate to ensure that the optimized value was in a reasonable range [37]. The CTLA-4 concentration in the model reflects that of non-healthy patients, as this was the only PK data available for optimization and validation of the ipilimumab PBPK model. Parameters related to antigen incorporation within the model are given in Table 3.

#### 2.3.2. PBPK Model Development for Ipilimumab

The PBPK model for ipilimumab utilized the structure for large molecules in GastroPlus described in Section 2.1 with the inclusion of endogenous IgG and the CTLA-4 antigen. The table of model input parameters for ipilimumab and CTLA-4 dynamics is given in Table 3. The initial fit of the model to a single dose of ipilimumab using both a 1 mg/kg and 3 mg/kg dose level from [9] in a patient population combining both the literature-derived parameter values along with the default GastroPlus parameters for an IgG molecule showed that the clearance phase was overpredicted. By optimizing only the degradation rate by the lysosomes (Kdeg), the model was able to match the observed terminal phase, but not the initial rapid decline. This indicated that the clearance was biphasic with an initial rapid clearance followed by a slower clearance phase. Therefore, in order to reproduce the observed profile, CTLA-4 blood concentration, internalization of the antibody–antigen complex (Kint), and degradation rate by the lysosomes (Kdeg) were optimized simultaneously. The initial CTLA-4 concentration value of 1.84 × 10^−6^ µmol/mL was based on an average of soluble CTLA-4 concentrations in various patient populations [34], optimized to 1.84 × 10^−5^ µmol/mL. Using CD28 as a surrogate for membrane-bound CTLA-4 (see Section 2.3.1), the optimized value is within the reported range for total CTLA-4 concentrations. Increasing the internalization of the antibody–antigen complex increases the clearance rate when ipilimumab binds to CTLA-4. By increasing the CTLA-4 concentration, this effect became more pronounced and drove the initial rapid clearance. The optimized Kdeg value accounted for the slower clearance terminal phase. The optimized values and final model parameters are given in Table 3 and Table 4. All default parameter values were used for the endogenous IgG present in the model. The quality and accuracy of the model predictions were judged by visual comparison of simulated and observed plasma concentration time profiles and by comparison of predicted and observed C_max_ and AUCs with simulated/observed ratios in the range of 0.8 to 1.25 considered accurate, 0.5 to 2 considered adequate, and ratios <0.5 or >2 (i.e., more than 2-fold error) deemed inadequate.

### 2.4. Nivolumab PBPK Model Development

#### 2.4.1. PD-1 Representation in Nivolumab PBPK Model

Nivolumab’s mechanism of action is to bind to PD-1 on the surface of immune cells to block the immune inhibition that occurs when PD-1 binds to PD-L1 on the cancer cell surface [5]. Nivolumab is thereby able to increase the anticancer action of immune cells. A representation of PD-1 as an antigen was incorporated into the model, introducing TMDD. PD-1 is expressed on immune cells that are primarily present in the lymph nodes [39]. However, within GastroPlus, antigens are not yet able to be represented in the lymph nodes. Therefore, an assumption was made that all PD-1 antigen was present only in the blood compartment and represented as soluble. An equation for PD-1 expression in cancer patients given in [39] was used to calculate PD-1 blood expression. Parameters associated with antigen–antibody binding dynamics and antigen degradation were taken from the literature [6,40]. Internalization of the antibody–antigen complex was calculated based on the decrease in PD-1 expression observed throughout treatment assuming a constant PD-1 synthesis and degradation rate [39,40]. The internalization rate was assumed to be the driving factor causing the decline in PD-1 expression. See Table 5 for final model input parameters for nivolumab and PD-1 dynamics.

#### 2.4.2. PBPK Model Development for Nivolumab

The PBPK model for nivolumab utilized the structure for large molecules in GastroPlus described in Section 2.1 with the inclusion of endogenous IgG and the PD-1 antigen. The summary of model input parameters for nivolumab is given in Table 6. The lysosomal degradation rate (Kdeg) was optimized to patient data and a separate value was optimized for the case of a single dose or multidose administration due to known accumulation of nivolumab after multiple doses [41,42]. The accumulation of nivolumab after multiple administrations is hypothesized to be driven by time-dependent clearance, potentially as a result of antibody cleavage by a tumor or consumption of the antibody as a source of protein in cases where the patient has an underlying metabolic illness such as cachexia in the case of cancer [41,42]. The exact mechanism is not known. Since time-dependent clearance is not able to be added in GastroPlus, the lysosomal degradation rate was decreased as a surrogate for this process. The endogenous IgG present in the model used the default GastroPlus parameter values. The quality and accuracy of the model predictions were judged by visual comparison of simulated and observed plasma concentration time profiles and by comparison of predicted and observed C_max_ and AUCs with simulated/observed ratios in the range of 0.8 to 1.25 considered accurate, 0.5 to 2 considered adequate, and ratios <0.5 or >2 (i.e., more than 2-fold error) deemed inadequate.

### 2.5. Linear Correlation Model Used to Predict Liver Concentration from Plasma Concentration as Validation for the PBPK Models

In the absence of measured liver concentrations after administration of the three monoclonal antibodies, a linear correlation model was used to validate the predicted liver concentrations from the simulated plasma concentrations using the PBPK model. The linear correlation model was developed by Shah et al. [5] using measured plasma and tissue-specific concentrations in mice, rats, monkeys, and humans after administration of various non-binding monoclonal antibodies. A subset of the mouse data was used to train the model and generate the antibody biodistribution coefficient for each tissue, while the remaining mouse data not used for the training, as well as rat, monkey, and human data, were used to validate the estimated coefficient. The tissue-specific antibody biodistribution coefficient is a multiplier to determine from measured plasma concentrations the estimated tissue concentration for non-binding monoclonal antibodies. In the case of the liver, across all species, the antibody biodistribution coefficient estimated using the mouse training data provided a reasonable prediction model for liver concentrations with the majority, and in some cases all, of the observed data points falling within 2-fold of the linear correlation model derived from the antibody biodistribution coefficient. The model was chosen to be used to validate liver interstitial concentration predictions from each of the three PBPK models.

## 3. Results

### 3.1. Infliximab PBPK Model

**Figure 3:**
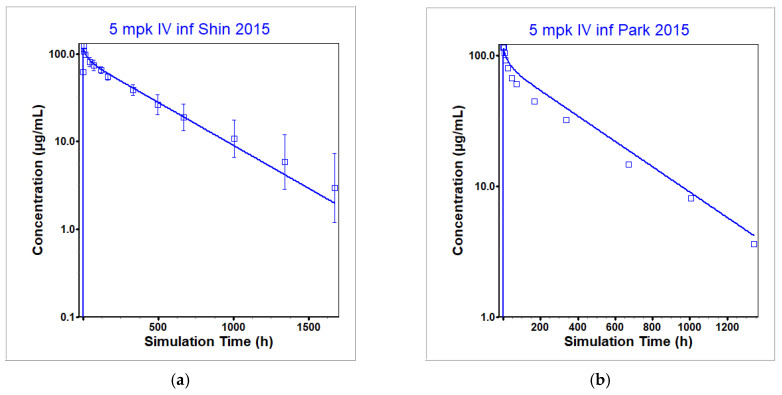
Observed and simulated plasma concentration time profiles for 5 mg/kg IV infusion of infliximab in healthy individuals without designation of ADA development. Final model includes increase in Koff in pH 6 to 800 per day. Squares are observed data, and the line represents the average model prediction. Data in [28] (**a**) were used for model optimization and data in [43] (**b**) were used for model validation.

The PBPK model for infliximab was generated in a step-wise process. The initial model prediction of the optimization dataset in Shin et al. [28] showed an underprediction of clearance (Figure 2). In order to fit to the observed data in [28], the rate at which infliximab unbinds from FcRn in pH 6 was increased from the default value of 500 per day to 800 per day. Higher unbinding led to increased clearance by lysosomes as the compound is not able to benefit from the protective effect of binding to FcRn to prevent lysosomal clearance. This single modification reproduced the observed data best across both available NHV clinical datasets [28,43] and, therefore, was chosen to be included in the final model. This modification was supported by in vitro data in [44]. The result of the optimization to [28] is given in Figure 3a with the model validation to a secondary dataset containing plasma concentration time profiles after an IV infusion in normal healthy individuals [43] in Figure 3b. Note that all infliximab concentrations were measured using an ELISA that quantified concentrations by measuring binding to TNF-α. Therefore, all measured concentrations are assumed to be unbound (not total) infliximab concentrations and were predicted as such within GastroPlus. However, in the case of infliximab at the dosing levels represented in the data and using the TNF-α concentrations reported in the literature, simulated total infliximab plasma concentrations equaled unbound plasma concentrations. See Table 7 (rows 1 and 2) for the simulated and observed PK parameters for the final model in healthy individuals. All simulated PK parameters (C_max_ and AUC) fell within bioequivalence limits (BE, 0.8–1.25) of the observed PK parameters for both optimization and validation datasets.

**Figure 4:**
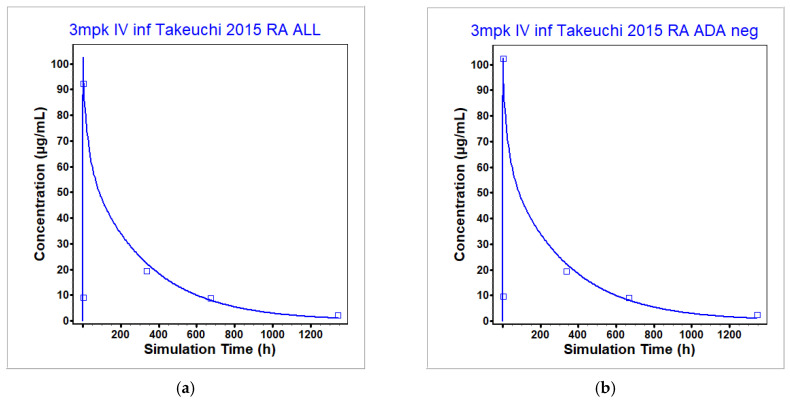

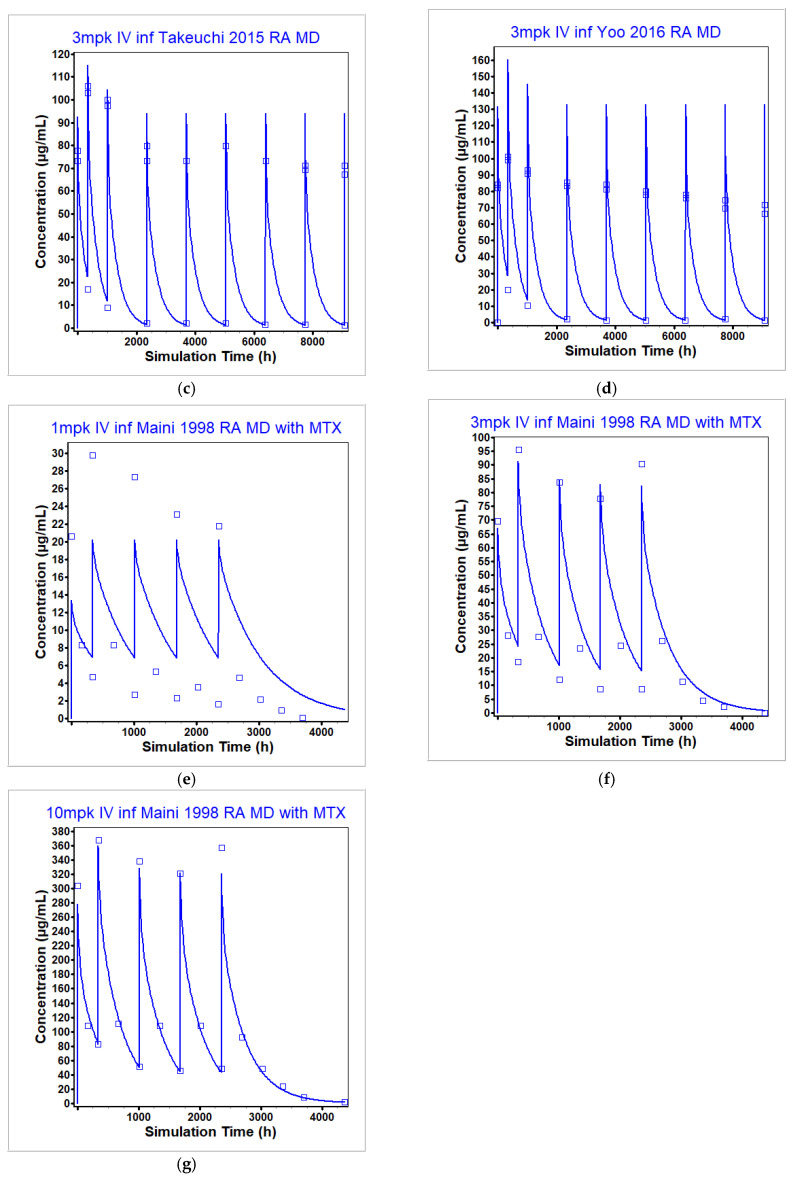
Observed and simulated plasma concentration time profiles used to validate the model for IV infusion infliximab administered in RA patients either without designated ADA development or who were measured as ADA negative. No changes to the model optimized in healthy patients without ADA designation were made to accommodate RA patients with the exception of an increase in plasma TNF-α levels to 30 pg/mL. Only (**b**) had a measured ADA status of negative. All others were not distinguished. (**a**) 3 mg/kg [45], (**b**) 3 mg/kg [45], (**c**) 3 mg/kg [45], (**d**) 3 mg/kg [47], (**e**) 1 mg/kg infliximab coadministered with methotrexate (MTX) [46], (**f**) 3 mg/kg infliximab coadministered with MTX [46], (**g**) 10 mg/kg infliximab coadministered with MTX [46]. The observed data are represented by the squares, and the average model prediction is represented by the line.

Once the model was optimized to healthy individuals, the model was then used for predictions in RA patients, as this is the patient population in which liver toxicity predictions will be made using the QST model BIOLOGXsym. The difference between healthy and RA patients in terms of the physiology in GastroPlus was an increase in TNF-α levels. Patients with inflammatory diseases such as RA are known to have higher TNF-α levels. Thilagar et al. [21] found that healthy patients have an average TNF-α concentration of 5.5 pg/mL, while RA patients can have an average concentration up to 30.5 pg/mL depending on the co-existence of other chronic conditions. RA patients in the GastroPlus model were characterized by this maximal increase in TNF-α levels. This change in TNF- α levels was sufficient to reproduce the observed data in RA patients without any other changes to the model optimized for NHV individuals. The model was validated against both single (Figure 4a,b) [45] and multidose administration of infliximab (Figure 4c–g) [45,46,47] in RA patients. Note that [46] contained multidose administration in RA patients with infliximab as a monotherapy and also in combination with methotrexate (MTX). Infliximab coadministered with MTX was chosen to validate the RA model for either ADA negative or non-reported ADA status as patients with monotherapy infliximab are more likely to develop ADAs [31] which can affect the PK of infliximab. Table 7 (rows 3 and 4) gives the simulated and observed PK parameters after single dose administration in RA patients. All simulated PK parameters fell within BE limits of the observed PK parameters after single dose administration in RA patients. For the multidose administrations, the plasma concentration time profiles were not characterized sufficiently to calculate the PK parameters. Overprediction of the C_max_ can be observed in some instances; however, as this is an IV infusion, this is likely attributable to incomplete information about the modeled population related to blood volume and not a reflection of model misspecification. Discrepancies at dose levels below the clinical dosing level (Figure 4e) are most likely attributable to incorrect characterization of the saturation of binding to TNF-α. Since liver toxicity predictions will not be made using sub-clinical dosing protocols, the rationale for model misfit to this dose level was not explored.

**Figure 5:**
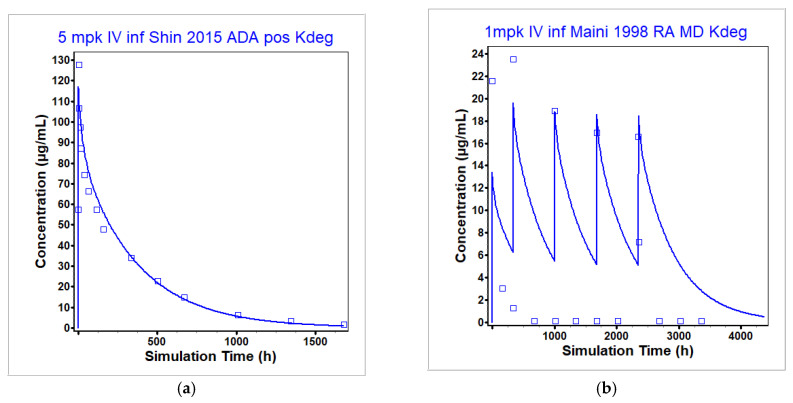

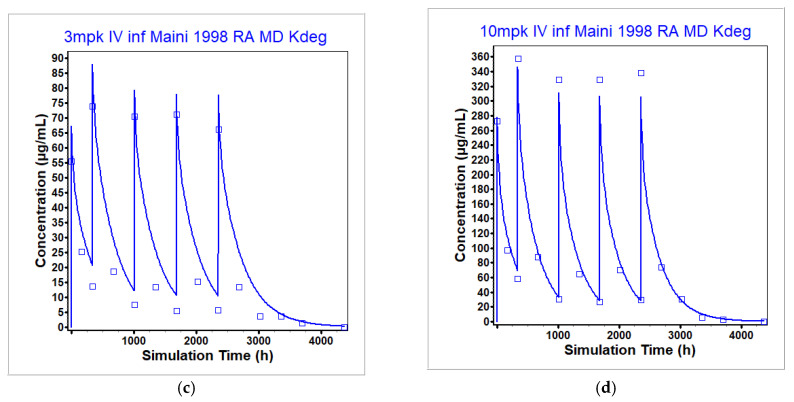
Simulated and observed plasma concentration time profiles used to fit (**a**) and validate (**b**–**d**) the infliximab model for IV infusion in individuals that developed ADAs. The degradation in lysosomes term (Kdeg) was increased to 12,500 per day to account for increased clearance compared to patients that did not develop ADAs. Only the healthy individuals in (**a**) tested positive for ADAs [28]. The ADA status of the RA patients represented in (**b**–**d**) at dose levels of 1, 3, and 10 mg/kg, respectively, with infliximab monotherapy was not reported. However, monotherapy infliximab administration is known to increase the probability of developing ADAs [31], and the ADA-calibrated model provided a better fit to the data compared to the model without increased clearance. The observed data are represented by the squares and the average model prediction is represented by the line.

The infliximab NHV model was also parameterized to account for the increased clearance that occurs in individuals that develop ADAs to infliximab as a result of the formation of an immune complex when infliximab binds to the ADAs [48]. See Section 2.2.3 for more details. Table 7 (rows 3 and 4) shows a comparison in the PK parameters in RA patients that never developed ADAs (row 4) with RA patients regardless of their ADA status (row 3). Patients that never developed ADAs have a higher AUC compared to all patients, indicative of a lower clearance rate. To account for increased clearance in patients that developed ADAs, the degradation term in the lysosomes was increased to 12,500 per day using the PK profile in healthy individuals that subsequently tested positive for ADAs measured in [28]. See Section 2.2.3 for more details. Table 7 (row 5) gives the corresponding simulated and observed PK parameters. All exposure parameters were within BE limits of the observed. Maini et al. [46] collected multidose administration data in RA patients administered infliximab as a monotherapy or in combination with MTX. They observed increased clearance in patients administered infliximab as a monotherapy compared to the combination therapy [46]. Patients that are administered infliximab in combination therapy are less likely to develop ADAs [31], and, indeed, the model without the increased clearance attributed to ADA development reproduced the combination therapy data the best (see Figure 4e–g). Increased degradation in the lysosomes reproduced the infliximab monotherapy data from [46] best (Figure 5b–d). Although the authors did not test for the presence of ADAs, it is likely that this was a contributing factor to the difference in infliximab PK between combination and monotherapy in this instance. Similar to the combination therapy data from the same authors at 1 mg/kg (Figure 4e), the sub-clinical dose level with infliximab monotherapy (1 mg/kg, Figure 5b) is not well predicted, potentially in part due to incorrect characterization of the saturation of binding of infliximab to TNF-α. Since the QST model will not be used to predict liver toxicity after sub-clinical dosing, further exploration was not performed to better represent this dose level.

### 3.2. Ipilimumab PBPK Model

**Figure 6:**
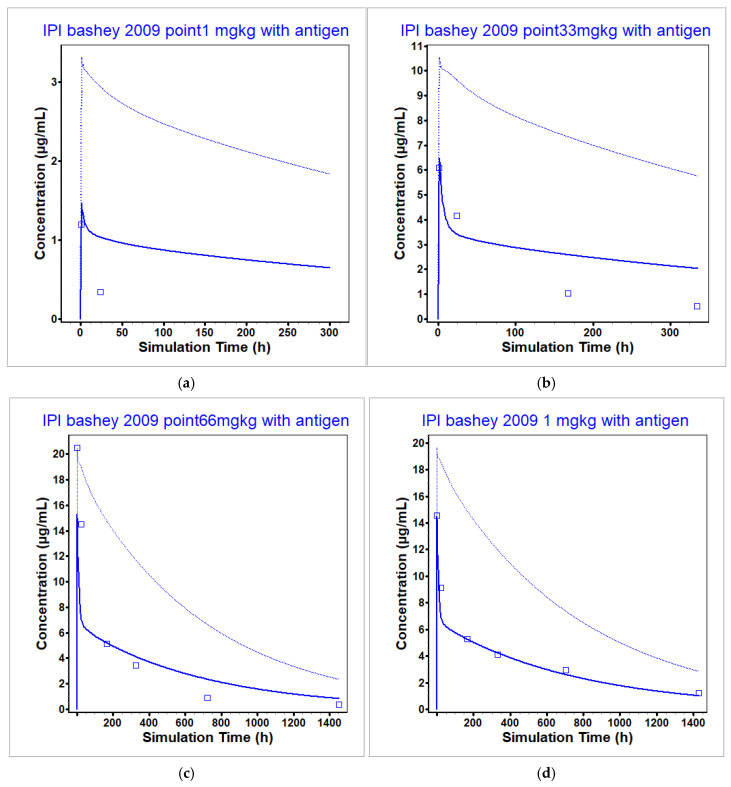
Ipilimumab simulated and observed PK profiles after single dose administrations at sub-clinical dose levels: (**a**) 0.1 mg/kg (validation), (**b**) 0.33 mg/kg (validation), (**c**) 0.66 mg/kg (validation), (**d**) 1 mg/kg (optimization) [9]. The observed data (unbound concentration) are represented by the squares and the average model prediction is represented by the line. The unbound plasma concentration of ipilimumab is given by the dark blue line and the total plasma concentration of ipilimumab is given by the light blue line.

**Figure 7:**
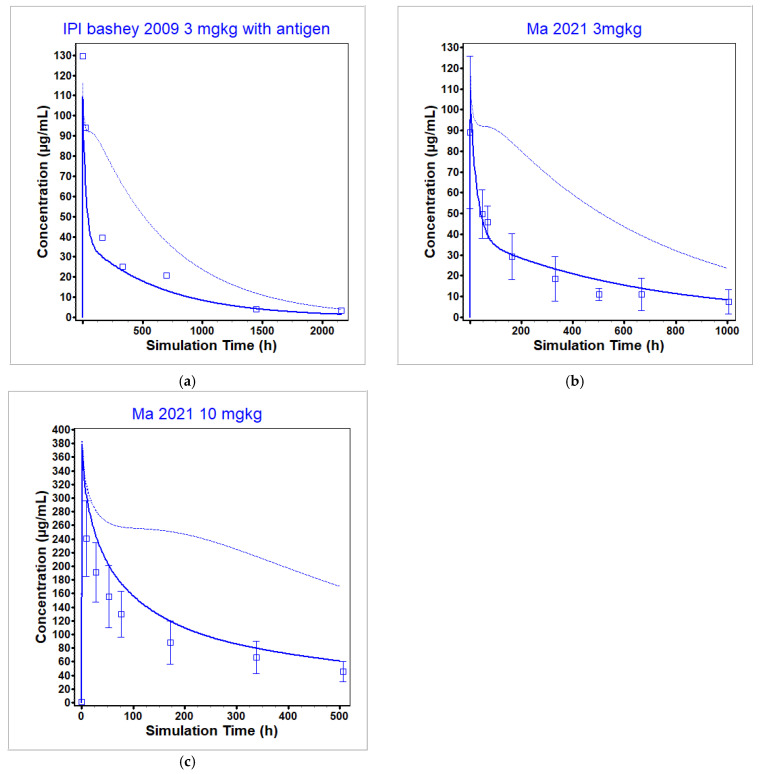
Ipilimumab simulated and observed PK profiles after single dose administrations at clinically relevant dose levels: (**a**) 3 mg/kg (optimization) [9], (**b**) 3 mg/kg (validation) [49], (**c**) 10 mg/kg (validation) [49]. The observed (unbound concentration) data are represented by the squares, and the average model prediction is represented by the line. The unbound plasma concentration of ipilimumab is given by the dark blue line and total plasma concentration of ipilimumab is given by the light blue line.

The ipilimumab PBPK model was optimized to a single dose of ipilimumab using both a 1 mg/kg and 3 mg/kg dose level from [9] in patients with relapse of malignancy after allogeneic hematopoietic cell transplantation, including patients with cancer and graft vs. host disease, and was validated across many dose levels both above and below the clinical dose level as well as via the use of multidose data in cancer patients. Note that all ipilimumab concentrations were measured using an ELISA that quantified concentrations by measuring binding to immobilized CTLA-4. Therefore, all measured concentrations are assumed to be unbound (not total) ipilimumab concentrations and were predicted as such within GastroPlus. In this case, unbound concentrations were different than total concentrations. The unbound concentrations were used to reproduce the observed data. The optimization and validation datasets are presented in Figure 6 and Figure 7. Figure 6 presents the model prediction under sub-clinical dose levels, while Figure 7 presents the model prediction under clinically relevant dose levels. Out of the seven datasets used for optimization (1 and 3 mg/kg from [9]) and validation (0.1, 0.33, and 0.66 mg/kg from [9] and 3 and 10 mg/kg from [49]), four had predicted C_max_ within BE limits with a total range of 0.75- to 1.56-fold of the observed values, and two had predicted AUC within BE limits with a total range of 0.69- to 1.44-fold of the observed values.

At the sub-clinical dose levels, the model was less able to reproduce the observed data compared to the clinical dosing levels. This may be attributed to less data informing the validity of the model or TMDD playing more of a role in the disposition of ipilimumab (i.e., the model is more sensitive to interpatient variability in CTLA-4 concentrations, implicitly driving the observed ipilimumab plasma concentration compared to the clinically relevant dose levels). Thus, poor model reproduction of the observed data at these dose levels can be attributed to the lack of data both in regards to observed ipilimumab plasma concentrations as well as patient-specific CTLA-4 levels. For example, re-optimization of the model within the reported range of CTLA-4 concentrations in a patient population was not able to capture the nonlinearity across dose. Additionally, it is possible that the interaction of ipilimumab with CTLA-4 was misspecified, with the current implementation not correctly capturing the nonlinearity in TMDD. However, without definitive information to the contrary, it is difficult to test this hypothesis. Since the model is to be used to predict liver concentrations after clinically relevant dosing levels, this was not further explored.

**Figure 8:**
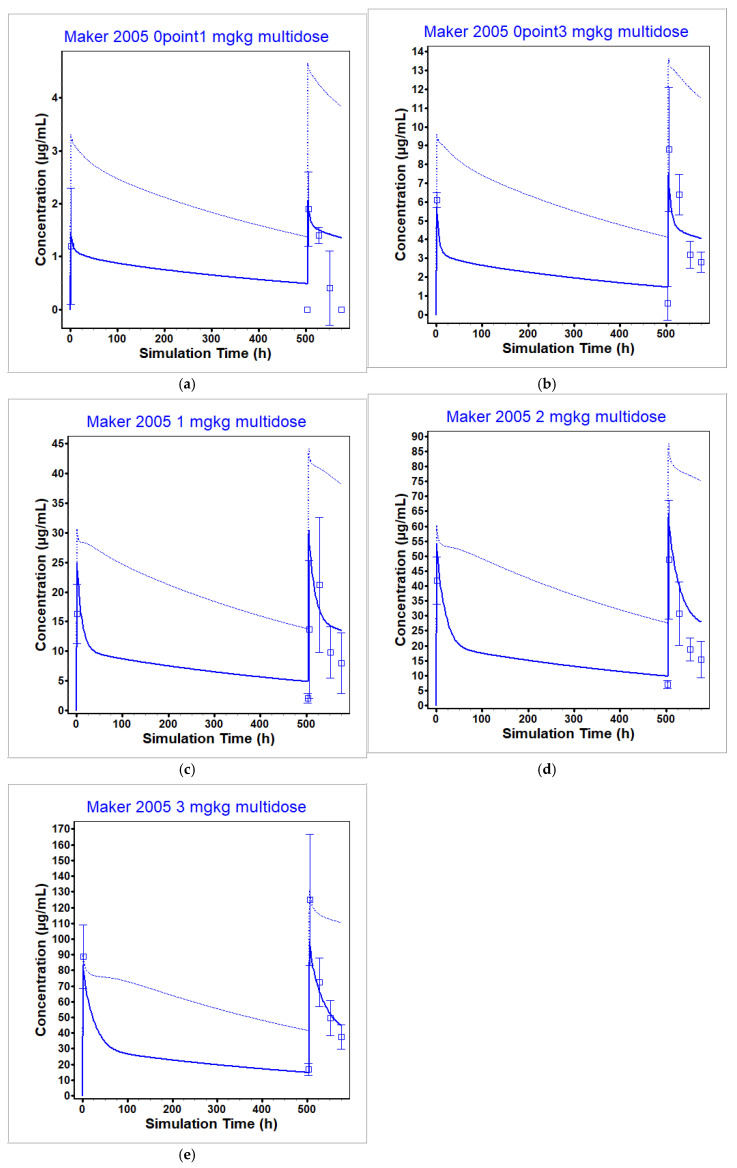
Ipilimumab PBPK model validated against multiple administration at dose levels: (**a**) 0.1 mg/kg, (**b**) 0.3 mg/kg, (**c**) 1 mg/kg, (**d**) 2 mg/kg, (**e**) 3 mg/kg [50]. The observed data (unbound concentration) are represented by the squares and the average model prediction is represented by the line. The unbound plasma concentration of ipilimumab is given by the dark blue line and the total plasma concentration of ipilimumab is given by the light blue line.

At the clinically relevant dose levels (3 and 10 mg/kg), the model was able to reproduce the observed data well within 2-fold (see Table 8). Further, the model was able to reproduce the observed ipilimumab plasma concentration after multiple administrations (Figure 8), although the model suffered from the same drawbacks as observed in the single dose administration (i.e., underprediction of clearance at sub-clinical dosing levels).

### 3.3. Nivolumab PBPK Model

The nivolumab PBPK model was optimized across three single administration dose levels (3 mg/kg, 10 mg/kg [51], and 480 mg [52]) in cancer and sepsis patients and validated against eight additional single dose administrations across a wide range of either at or below clinically relevant dose levels [51,53,54] in cancer and sepsis patients. A separate lysosomal degradation rate was optimized in the case of multidose administration [55] to account for the known time-dependent clearance of nivolumab [41,42]. Note that all nivolumab concentrations were measured using an ELISA that quantified concentrations by measuring binding to immobilized PD-1. Therefore, all measured concentrations were assumed to be unbound (not total) nivolumab concentrations and were predicted as such within GastroPlus. However, in the case of nivolumab at the dosing levels represented in the data and the PD-1 concentration reported in the literature, total nivolumab plasma concentrations equaled unbound nivolumab plasma concentrations. A sensitivity analysis for PD-1 levels showed that within physiological range, unbound and total nivolumab concentrations are equal (see Figure S2 in the Supplementary Materials).

**Figure 9:**
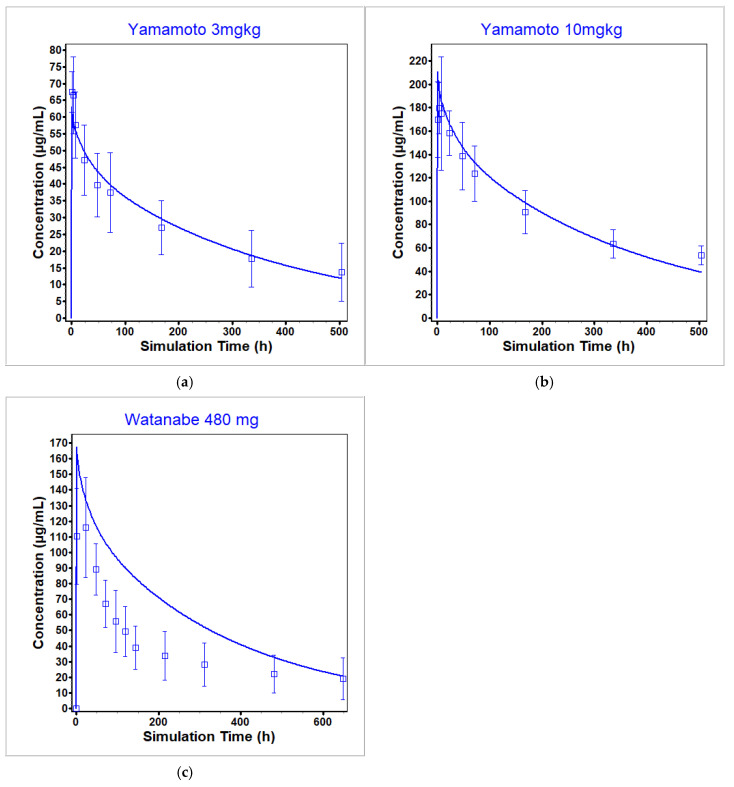
Optimization datasets used for the nivolumab PBPK model at dose levels (**a**) 3 mg/kg [51], (**b**) 10 mg/kg [51], and (**c**) 480 mg [52]. The observed data are represented by the squares, and the average model prediction is represented by the line.

**Figure 10:**
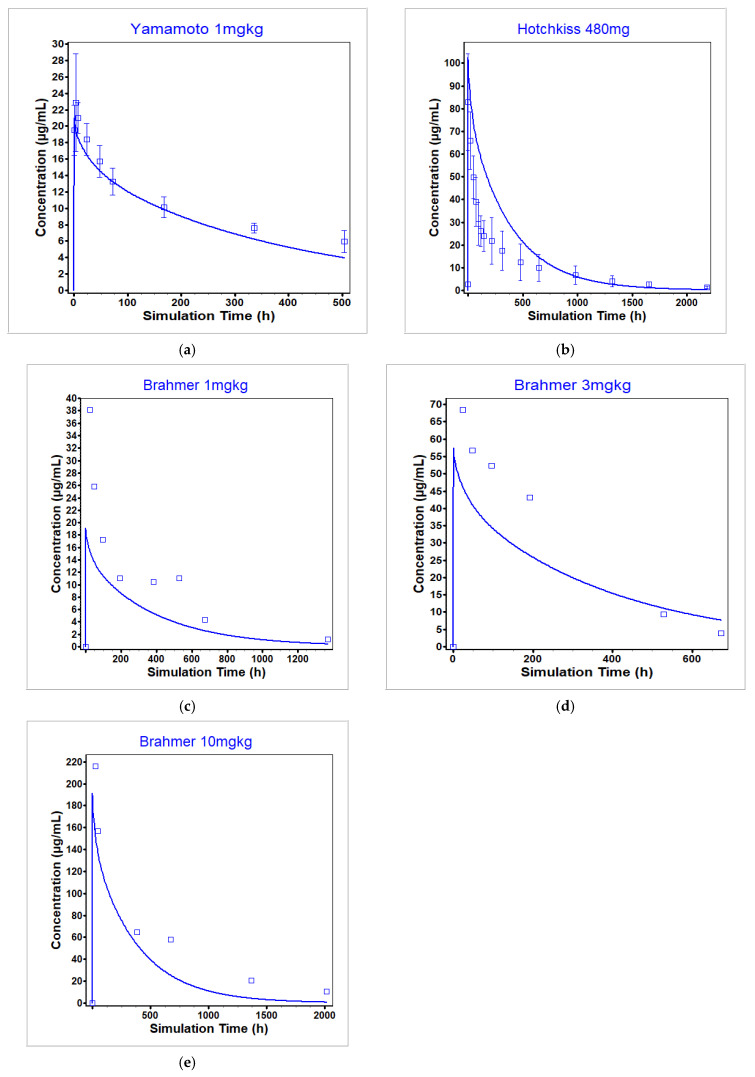
Validation datasets used for the nivolumab PBPK model at dose levels (**a**) 1 mg/kg [51], (**b**) 480 mg [54], (**c**) 1 mg/kg [53], (**d**) 3 mg/kg [53], and (**e**) 10 mg/kg [53]. The observed data are represented by the squares and the average model prediction is represented by the line.

**Figure 11:**
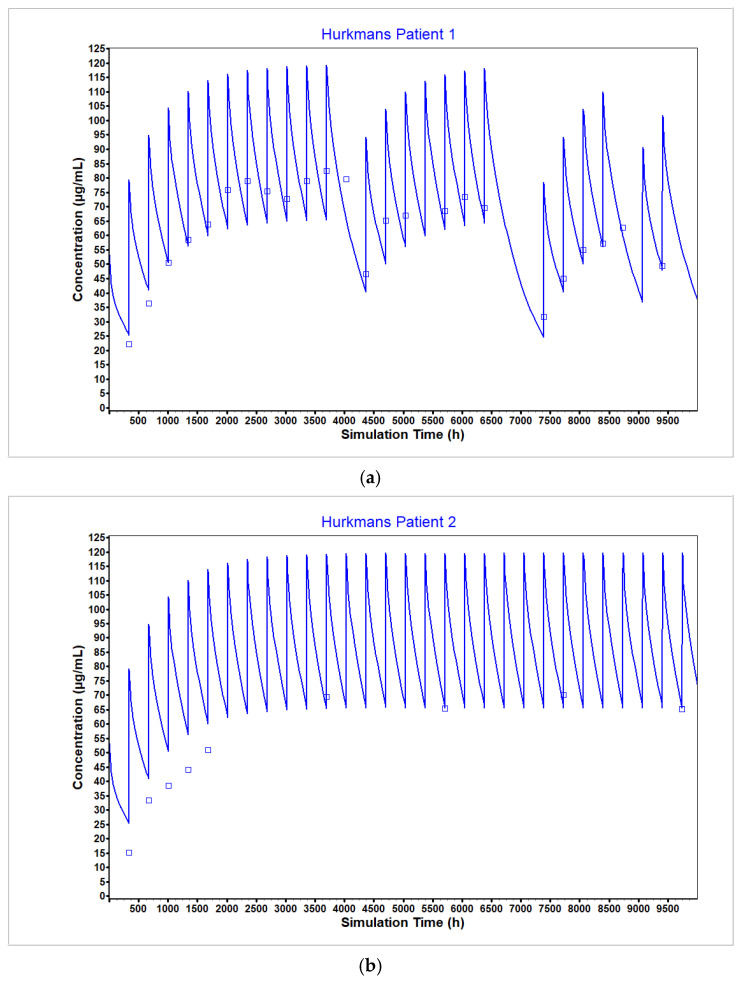
Model optimization and validation to the multidose administration profiles in [55]. The lysosomal degradation rate (Kdeg) was optimized using the plasma concentration time curve from patient 1 (**a**) and validated using the plasma concentration time curve from patient 2 (**b**). Decreasing the lysosomal degradation rate compared to single dose administration is used to capture the known time-dependent decrease in nivolumab clearance. The observed data are represented by the squares and the average model prediction is represented by the line.

Figure 9 shows the fit of the model to the datasets used for optimization, Figure 10 shows the fit of the model to the datasets used for single dose administration validation, and Figure 11 shows the fit of the model to the multidose administration data. Out of the 11 single dose datasets used for optimization and validation, 6 had a predicted C_max_ within BE limits with a total range of 0.5- to 2.17-fold of observed values and 3 had a predicted AUC within BE limits with a total range of 0.5- to 1.87-fold of the observed values. For the multidose fitting dataset, 67% of the simulated C_troughs_ fell within BE limits with a total range of 0.77- to 2.05-fold of the observed values. For the multidose validation dataset, 33% of the simulated C_troughs_ fell within BE limits with a total range of 1.01- to 2.7-fold of the observed values. See Table 9 for the ratio of simulated to observed exposure PK parameters for the single dose datasets.

The model was able to reproduce the observed data from the Yamamoto study [51] in cancer patients across dose levels. However, at the same dose level also in cancer patients, the model was not able to recapitulate the observed data from the Brahmer study [53]. While both studies included patients with refractory solid tumors, the difference may be attributable to the differences in cancer type included in each study. The Yamamoto dataset was chosen for optimization as more comprehensive data were available (i.e., mean and standard deviation) as compared to the single mean data points reported in the Brahmer study. Additionally, the model was not able to capture either the Watanabe [52] or Hotchkiss [54] studies well, although the Watanabe study was included in model optimization. Both the Watanabe and Hotchkiss studies involve patients with sepsis and not cancer. Therefore, this difference may be attributable to differences in PD-1 levels between the two patient types, as PD-1 levels used in the model were estimated using cancer patient data.

The lysosomal degradation rate for multidose administration was optimized to a lower value compared to that for a single dose administration to achieve the observed reduced clearance (Figure 11). As expected, this change led to overestimation of plasma concentration as measured by C_trough_ for early time points when the time-dependent clearance might not yet have changed. However, at steady state, the updated model matched the observed plasma measurements. It should be noted that only two individual plasma concentration time profiles were used to optimize and validate this parameter change. It is not clear without additional validation data how well the value translates across dose levels.

### 3.4. Predicted Interstitial Liver Concentrations

The PBPK models developed for infliximab, ipilimumab, and nivolumab based on optimization to plasma concentration time data were used to predict liver interstitial concentrations after administration of clinically relevant doses at which liver toxicity has been reported. This was used to inform liver exposure in the QST platform BIOLOGXsym. Liver concentrations after administration of these monoclonal antibodies were not available in the literature to validate the models’ predictions. However, Shah et al. [5] developed a linear correlation model between mAb plasma concentration and liver concentration validated across many different species, including humans, using experimentally obtained plasma and tissue concentrations. See Section 2.5 for more details. This previously developed correlation model was used to validate the predicted liver interstitial concentrations given simulated plasma concentrations from the PBPK model after administration of each of the three large molecules.

The antibody biodistribution coefficient (ABC) from the liver estimated from Shah et al. [5] was 12.1% with a coefficient of variation of 3.75%. This indicates that liver concentrations are estimated to be approximately 12.1% of plasma concentrations after administration of non-binding antibodies (i.e., antibodies that do not bind to any target). The liver concentrations resulting from simulating a single clinical dose of 5 mg/kg for infliximab, 3 mg/kg for ipilimumab, and 3 mg/kg for nivolumab ranged from 10% of plasma concentrations (occurring right after T_max_) to 23% of plasma concentrations. Although the proportion of drug in the liver compared to plasma was predicted to be higher once the differential has reached a steady state, the value was within 2-fold of the estimated ABC for the liver. This provides validation of the predicted liver concentration from the PBPK models after administration of the monoclonal antibodies.

## 4. Discussion

Three PBPK models for the monoclonal antibodies infliximab, ipilimumab, and nivolumab were optimized and validated using clinically observed plasma concentration time profiles across many different dose levels. The majority of the parameter values came from the literature, with only a few parameters optimized in each model. Each model incorporated a binding antigen (TNF-α in the case of infliximab, CTLA-4 in the case of ipilimumab, and PD-1 in the case of nivolumab). The PBPK model for infliximab was calibrated for predictions in both healthy and RA-specific patients, while those for ipilimumab and nivolumab were specific to non-healthy patients, as clinical PK data were only available in patient populations. In all three cases, the non-healthy patient population was characterized by increased levels of the drug’s target antigen compared to a typical range in a healthy individual. Patient variability in antigen concentration was not directly explored. However, simulations of an average individual exploring a range of physiological values for antigen concentrations did not show any difference in the predicted plasma concentration time profile in the case of infliximab and nivolumab (Figures S1 and S2 in the Supplementary Materials, respectively). Target antigen concentrations of CTLA-4 were shown to have an effect on the PK of ipilimumab. However, due to the lack of data in patient variation of CTLA-4, this was not explored in the model.

The increased clearance necessary (compared to the default parameterization for IgGs) to reproduce observed behavior of infliximab in healthy individuals came from increasing the unbinding rate to the FcRn in pH 6. It was found in Arora et al. [44] that in the absence of TNF-α, infliximab poorly binds to the FcRn. This led to the hypothesis that either a decreased binding rate to the FcRn or an increased unbinding rate to the FcRn at pH 6 could be the mechanism to induce increased clearance. Ultimately, based on the fit across clinical datasets (5 mg/kg dose level from Shin et al. [28] used for optimization and 5 mg/kg dose level from Park et al. [43] used for validation) measured by the ratio of simulated to observed exposure PK parameters, increasing the unbinding rate to the FcRn in pH 6 from 500 to 800 per day was selected as the modification necessary to reproduce the observed data. The further increased clearance observed in patients that developed ADAs after receiving infliximab was accounted for in the model by increasing the lysosomal clearance rate compared to patients that did not develop ADAs. Clearance in the ipilimumab model appeared to be biphasic with an initial rapid decline followed by a slower one. TMDD appeared to be driving the faster initial clearance phase, while degradation by the lysosomes was responsible for the later slower clearance phase. Optimizing CTLA-4 concentrations beyond what was observed in the literature to account for unmeasured membrane bound concentrations as well as internalization of the ipilimumab–CTLA-4 complex reproduced the initial clearance phase, and optimizing the lysosomal degradation reproduced the later clearance phase. Decreased clearance that had been observed with multiple doses of nivolumab administration was incorporated with decreased lysosomal clearance in the case of multidose administration compared to administration of a single dose.

All three PBPK models were able to reproduce observed plasma concentration time profiles, mostly within 2-fold of the observed, and most within BE limits. This gives confidence that the model is properly specified for the three large molecules. For each of the three PBPK models, the predicted range of liver concentration as a percent of plasma concentration was between 10% and 23% depending on time after dose. The model’s predicted liver concentration fell within 2-fold of what was expected from the previously developed linear correlation model calibrated with experimental data [5]. This provides further evidence that the models were properly specified and can make reasonable predictions of both plasma and hepatic concentrations given IV administrations of infliximab, ipilimumab, and nivolumab in the relevant populations.

To the authors’ knowledge there are no existing ipilimumab or nivolumab PBPK models in the literature. A few publications were found detailing infliximab PBPK model development and application [56,57,58]. However, all three publications applied their models to different populations than what was considered in this paper (i.e., predictions in pediatric populations or predictions in inflammatory bowel patient populations). Further, in Chang et al. [56], a de novo PBPK model was built which had a one compartment endosomal subspace, whereas GastroPlus uses a three compartment endosomal subspace to account for pH specific binding of the antibody to FcRn, and so the two models are not directly comparable.

A limitation of the models’ assumption of target antigen residing only in the blood compartment is that effects of the antigen on the drug in the tissue are not well captured. As an example, TNF-α is known to be present in tissues such as the liver, especially in RA patients [59]. Clearance of infliximab in the tissue via internalization of the antigen–antibody complex is not explicitly represented since TNF-α is not represented in the tissues. However, since the unbinding rate of the antibody to FcRn in pH 6 was optimized (which leads to higher clearance via lysosomal degradation), in the case of infliximab and TNF-α, this optimization may implicitly include contributions from antigen-antibody clearance.

One limitation of using the Shah linear correlation model to validate the liver interstitial concentrations predicted from the PBPK models is that the correlation was developed using non-binding antibodies. Infliximab, ipilimumab, and nivolumab all bind to a target antigen and that process is incorporated within the PBPK model. In the case of infliximab and nivolumab across all modeled dose levels using reported antigen concentrations found in the literature, total and unbound drug concentrations in the blood (the location of the binding antigen) were equal, indicating that TMDD does not affect the PK of the compound and so the linear correlation model results can be adequately used to predict liver interstitial concentrations from plasma concentrations in these models. However, in the case of ipilimumab, total and unbound drug concentrations in the plasma were not the same, and in fact, antigen concentration was one parameter that required optimization in the model. This would imply that TMDD is going to have more of an effect on the PK of the compound. Likely, the linear correlation model would predict higher liver interstitial concentrations because it is not accounting for the effect of binding to the antigen: due to its size, the antigen–antibody complex may not be able to enter the liver, reducing the hepatic concentration of the drug. In the case of toxicity predictions, a higher predicted drug concentration at the site of action may result in a more conservative toxicity prediction, while in the case of efficacy predictions the opposite would be true. Therefore, the results from the linear correlation model need to be applied with more caution when predicting liver interstitial concentrations of ipilimumab from plasma concentrations.

These three PBPK models can be used in a wide variety of clinical applications, including dose predictions of infliximab in RA patient populations, dose predictions of ipilimumab in patients with malignancy after allogeneic hematopoietic cell transplantation, including patients with cancer and graft vs. host disease, and dose predictions of nivolumab in cancer patients, as the models were calibrated to data after drug administration in these patient populations. Additionally, these models can be modified for pediatric dose predictions by changing the underlying physiology from one representative of adults to one representative of pediatrics. Slight recalibration and validation would be necessary, but could ultimately be conducted in order to change the population in which these models could be used to make predictions, e.g., changing the relevant patient population from RA to irritable bowel syndrome for infliximab dosing. However, one limitation of these models is that the patient population within the model differs only from a healthy population in concentration of the target antigen. Other disease effects that may be relevant to the compound’s PK (such as disease-specific changes to physiology) are not included in the model. Due to the software platform’s flexibility, these changes can be added if the necessary data are available for parameterization.

The hepatic exposure predictions from each of these models will be used to inform toxicity predictions in the QST software platform BIOLOGXsym in conjunction with in vitro data about the drug’s capacity to target known liver toxicity mechanisms. It is crucial to have an accurate estimate of drug exposure in the liver in order to make these toxicity predictions. Developing accurate PBPK models informed by data is one way in which to achieve this end.

## Figures and Tables

**Table 1:** PBPK model parameter input values for infliximab and TNF-α dynamics.

Model Parameter	Unit	Value	Source
Infliximab MW	g/mol	149,100	[7]
TNF-α MW	g/mol	17,000	[23]
TNF-α degradation	1/day	16.6	Based on value used in BIOLOGXsym to promote stable dynamics
TNF-α NHV blood expression	µmol/g-tissue	1.32 × 10^−10^	[21,24,25]
TNF-α RA blood expression	µmol/g-tissue	7.94 × 10^−10^	[21]
Kon (antibody-antigen)	1/µM/day	8372.16	[26]
Koff (antibody-antigen)	1/day	75.95	[26]
Kint (internalization rate of the antibody-antigen complex)	1/day	0.089	[27]

**Table 2:** Final exogenous IgG model parameters for the infliximab PBPK model.

Model Parameter	Value	Source
Vascular reflection coefficient	0.95	Default
Lymphatic reflection coefficient	0.2	Default
Endosomal uptake rate (1/day)	12	Default
Recycle rate (1/day)	12	Default
Vascular rate fraction	0.72	Default
Kon, FcRn (pH 7.4) (1/µM/day)	90	Default
Kon, FcRn (pH 6.0) (1/µM/day)	8000	Default
Koff, FcRn (pH 7.4) (1/day)	3 × 10^4^	Default
Koff, FcRn (pH 6.0) (1/day)	800	Optimized
Kdeg (1/day)	1 × 10^4^	Default

**Table 3:** PBPK model input values for ipilimumab and CTLA-4 dynamics.

Parameter	Unit	Value	Source
Ipilimumab MW	g/mol	148,000	[8]
CTLA-4 MW	g/mol	25,000	[32]
CTLA-4 decay rate	1/day	6.1235	[33]
CTLA-4 blood concentration	µmol/g-tissue	1.84 × 10^−5^	Optimized
Kon (antigen-antibody)	1/µM/day	33,091.2	[38]
Koff (antigen-antibody)	1/day	601.344	[38]
Kint (internalization rate of the antibody-antigen complex)	1/day	0.04	Optimized

**Table 4:** Final exogenous IgG model parameters for the ipilimumab PBPK model.

Model Parameter	Value	Source
Vascular reflection coefficient	0.95	Default
Lymphatic reflection coefficient	0.2	Default
Endosomal uptake rate (1/day)	12	Default
Recycle rate (1/day)	12	Default
Vascular rate fraction	0.72	Default
Kon, FcRn (pH 7.4) (1/µM/day)	90	Default
Kon, FcRn (pH 6.0) (1/µM/day)	8000	Default
Koff, FcRn (pH 7.4) (1/day)	3 × 10^4^	Default
Koff, FcRn (pH 6.0) (1/day)	500	Default
Kdeg (1/day)	6000	Optimized

**Table 5:** PBPK model input parameter values for nivolumab and PD-1 dynamics.

Parameter	Unit	Value	Source
Nivolumab MW	g/mol	143,600	[10]
PD-1 MW	g/mol	29,289.06	[39]
PD-1 decay rate	1/day	0.33648	[40]
PD-1 blood concentration	µmol/g-tissue	1.04 × 10^−9^	[39]
Kon (antigen-antibody)	1/µM/day	21,685	[6,40]
Koff (antigen-antibody)	1/day	66.3552	[6,40]
Kint (internalization rate of the antibody-antigen complex)	1/day	0.4474	Calculated using [39,40]

**Table 6:** Final exogenous IgG model parameters for the nivolumab PBPK model.

Model Parameter	Value	Source
Vascular reflection coefficient	0.95	Default
Lymphatic reflection coefficient	0.2	Default
Endosomal uptake rate (1/day)	12	Default
Recycle rate (1/day)	12	Default
Vascular rate fraction	0.72	Default
Kon, FcRn (pH 7.4) (1/µM/day)	90	Default
Kon, FcRn (pH 6.0) (1/µM/day)	8000	Default
Koff, FcRn (pH 7.4) (1/day)	3 × 10^4^	Default
Koff, FcRn (pH 6.0) (1/day)	500	Default
Kdeg (1/day)	20,000	Optimized (single dose)
Kdeg (1/day)	11,000	Optimized (multidose)

**Table 7:** Simulated (Sim) and observed (Obs) PK parameters for all datasets used in optimization and validation of infliximab model after IV infusion. Simulated data refers to results from the model, and observed data refers to the data collected in the literature. The model calibrated for the RA patient population has a higher TNF-α concentration as compared to the model for healthy individuals. The model calibrated for the individuals that were ADA positive has an increased clearance as compared to the models for ADA negative patients, populations without information on ADA status (NA), or populations where PK profiles were grouped together (Both).

Study	Health Status	ADA Positive or Negative	Dose [mg/kg]	PK Parameter	Obs	Sim	Sim/Obs
Shin 2015 [28]	Healthy	Both	5	C_max_ [µg/mL]	124.1	117.3	0.95
				AUC_0-t_ [µg·h/mL]	38,550	38,070	0.99
				AUC_0-inf_ [µg·h/mL]	39,990	38,960	0.97
Park 2015 [43]	Healthy	NA	5	C_max_ [µg/mL]	116.7	114.9	0.98
				AUC_0-t_ [µg·h/mL]	30,820	36,720	1.19
				AUC_0-inf_ [µg·h/mL]	32,320	38,600	1.19
Takeuchi 2015 [45]	RA	Both	3	C_max_ [µg/mL]	108	102.4	0.95
				AUC_0-t_ [µg·h/mL]	21,900	21,020	0.96
Takeuchi 2015 [45]	RA	Negative	3	C_max_ [µg/mL]	111	102.4	0.92
				AUC_0-t_ [µg·h/mL]	24,700	21,020	0.85
Shin 2015 [28]	Healthy	Positive	5	C_max_ [µg/mL]	124.1	117.3	0.95
				AUC_0-t_ [µg·h/mL]	31,870	32,030	1.01
				AUC_0-inf_ [µg·h/mL]	32,620	32,370	0.99

**Table 8:** Simulated (Sim) and observed (Obs) PK parameters for all single dose datasets used for optimization and validation of the ipilimumab PBPK model. Simulated data refers to results from the model and observed data refers to the data collected in the literature.

Source	Dose [mg/kg]	Obs C_max_ [µg/mL]	Sim C_max_ [µg/mL]	Sim/Obs	Obs AUC_0-t_ [µg·h/mL]	Sim AUC_0-t_ [µg·h/mL]	Sim/Obs
Bashey 2009 [9]	0.1	1.195	1.38	1.15	NA ^1^	25.14	NA
Bashey 2009 [9]	0.33	6.094	6.47	1.06	626.2	900.58	1.44
Bashey 2009 [9]	0.66	20.48	15.3	0.75	3766.7	4104.44	1.09
Bashey 2009 [9]	1	14.56	14.51	1.00	4871.1	4288.13	0.88
Bashey 2009 [9]	3	129.5	109.61	0.85	37,790	25,991.08	0.69
Ma 2021 [49]	3	88.96	109.61	1.23	19,370	25,991.08	1.34
Ma 2021 [49]	10	241	376.13	1.56	45,460	58,740.24	1.29

^1^ Only two measured concentrations.

**Table 9:** Simulated (Sim) and observed (Obs) PK parameters for all single dose datasets used for optimization and validation of the nivolumab PBPK model. Simulated data refers to results from the model and observed data refers to the data collected in the literature.

Source	Dose [mg/kg]	Obs C_max_ [µg/mL]	Sim C_max_ [µg/mL]	Sim/Obs	Obs AUC_0-t_ [µg·h/mL]	Sim AUC_0-t_ [µg·h/mL]	Sim/Obs
Yamamoto 2017 [51]	1	22.9	21.0	0.92	4980	4390	0.88
Yamamoto 2017 [51]	3	67.4	63.1	0.94	1.28 × 10^4^	1.32 × 10^4^	1.03
Yamamoto 2017 [51]	10	180	211	1.17	4.37 × 10^4^	4.39 × 10^4^	1.00
Watanabe 2020 [52]	480 mg	116	167	1.44	2.41 × 10^4^	3.83 × 10^4^	1.59
Hotchkiss 2019 [54]	480 mg	82.8	102	1.23	2.1 × 10^4^	3.08 × 10^4^	1.47
Brahmer 2010 [53]	1	38.1	19.1	0.5	1.03 × 10^4^	5550	0.54
Brahmer 2010 [53]	3	68.4	57.4	0.84	1.93 × 10^4^	1.42 × 10^4^	0.74
Brahmer 2010 [53]	10	216	191	0.88	9.94 × 10^4^	5.69 × 10^4^	0.57

## Data Availability

The original contributions presented in this study are included in the article/Supplementary Material. Further inquiries can be directed to the corresponding authors.

## Supplementary Materials

The following supporting information can be downloaded at: https://www.mdpi.com/article/10.3390/pharmaceutics17030372/s1, Section S1. Biologics Module Equations. Section S2. Sensitivity Analysis on TNF-α Levels in the Infliximab PBPK Model. Section S3. Sensitivity Analysis on PD-1 Levels in the Nivolumab PBPK Model.
