# Supplementary material for: Physiologically Based Pharmacokinetic Models for Infliximab, Ipilimumab, and Nivolumab Developed with GastroPlus to Predict Hepatic Concentrations
Source: Pharmaceutics. 2025 Mar 14;17(3):372. doi: 10.3390/pharmaceutics17030372 (PMC11945841; doi:10.3390/pharmaceutics17030372)
Supplement: Supplementary file 1 [file pharmaceutics-17-00372-s001.zip › pharmaceutics-3503065-supplementary.pdf]

Supplementary Material

Section S1. Biologics Module Equations

Unless otherwise specified the variables have the following definition:

σ_v is the vascular reflection coefficient

σ_L is the lymph reflection coefficient

C_v is the antibody concentration in the vascular space

C_i is the antibody concentration in the interstitial space

L is the tissue lymph flow

$F_{leakage}$ is the relative vascular leakage

R1 is the total endocytosis uptake rate by endothelial cells

R2 is the recycling rate back to the vascular and interstitial spaces

FR is the fraction of the total endocytosis uptake rate coming from the vascular space (remainder of the endocytosis uptake rate is from the interstitial space)

τ is the transit time of each endosomal subspace

K_{on} (7.4) is the binding rate constant of antibody to FcRn in pH 7.4

K_{off} (7.4) is the dissociation rate constant of antibody to FcRn in pH 7.4

K_{on} (6.0) is the binding rate constant of antibody to FcRn in pH 6.0

K_{off} (6.0) is the dissociation rate constant of antibody to FcRn in pH 6.0

K_{deg} is the lysosomal degradation rate constant

V_{endo-1}, V_{endo-2}, V_{endo-3} are the volumes in the individual endosomal subspaces

C^f_{endo-1}, C^f_{endo-2}, C^f_{endo-3} are the unbound (free) antibody concentrations in the individual endosomal subspaces

C^b_{endo-1}, C^b_{endo-2}, C^b_{endo-3} are the concentrations of FcRn-bound antibody in individual endosomal subspaces

$FcRn^f_{endo-1}$, $FcRn^f_{endo-2}$, $FcRn^f_{endo-3}$ are the free FcRn concentrations in individual endosomal subspaces

$K_{syn,antigen}$ is the synthesis rate of the antigen

$K_{deg,antigen}$ is the degradation rate of antigen

$K_{int,TMD}$ is the internalization rate of antigen-antibody complex

$K_{on,antigen}$ is the association rate constant between antigen and antibody

$K_{off,antigen}$ is the dissociation rate constant between antigen and antibody

$C_{antigen}$ is the antigen concentration in the interstitial space

V_i is the volume of the interstitial space

C^f_i is the free antigen concentration in the interstitial space

C^b_i is the bound antigen-antibody concentration in the interstitial space

Equation S1. Vascular to interstitial and interstitial to lymph transfer rates

$$\textit{Vascular-to-Interstitial-Transfer-Rate} = (1 - \sigma_v) \times C_v \times L$$

$$\textit{Interstitial-to-Lymph-Transfer-Rate} = (1 - \sigma_L) \times C_i \times L$$

Equation S2. Vascular reflection coefficient

$$\sigma_v = \begin{cases} \sigma_{v,muscle} \times f_{leakage}; & f_{leakage} < 1 \\ \sigma_{v,muscle} + (1 - \sigma_{v,muscle}) \times (f_{leakage} - 1); & f_{leakage} \geq 1 \end{cases}$$

Equation S3. Antibody endocytosis rate

$$\textit{mAb-Endocytosis-Rate} = R1 \times FR \times C_v \times V_{endo} + R1 \times (1 - FR) \times C_i \times V_{endo}$$

Equation S4. Free antibody in the first endosomal subspace

$$\textbf{Free mAb: } V_{endo-1}\frac{dC^{f}_{endo-1}}{dt} = R1 \times FR \times C_v \times V_{endo} + R1 \times (1-FR) \times C_i \times V_{endo} - K_{on(7.4)} \times FcRn^{f}_{endo-1} \times C^{f}_{endo-1} \times V_{endo-1} + K_{off(7.4)} \times C^{b}_{endo-1} \times V_{endo-1} - \frac{1}{\tau} \times C^{f}_{endo-1} \times V_{endo-1}$$

Equation S5. Bound antibody in the first endosomal subspace

$$\textbf{Bound mAb: } V_{endo-1}\frac{dC^{b}_{endo-1}}{dt} = R2 \times C^{B}_{endo-3} \times V_{endo} + K_{on(7.4)} \times FcRn^{f}_{endo-1} \times C^{f}_{endo-1} \times V_{endo-1} - K_{off(7.4)} \times C^{b}_{endo-1} \times V_{endo-1} - \frac{1}{\tau} \times C^{b}_{endo-1} \times V_{endo-1}$$

Equation S6. Free FcRn in the first endosomal subspace

$$\textbf{Free FcRn: } V_{endo-1}\frac{dFcRn^{f}_{endo-1}}{dt} = R2 \times FcRn^{f}_{endo-3} \times V_{endo} - K_{on(7.4)} \times FcRn^{f}_{endo-1} \times C^{f}_{endo-1} \times V_{endo-1} + K_{off(7.4)} \times C^{B}_{endo-1} \times V_{endo-1} - \frac{1}{\tau} \times FcRn^{f}_{endo-1} \times V_{endo-1}$$

Equation S7. Free antibody in the second endosomal subspace

$$\textbf{Free mAb: } V_{endo-2}\frac{dC^{f}_{endo-2}}{dt} = \frac{1}{\tau} \times C^{f}_{endo-1} \times V_{endo-1} - K_{on(6.0)} \times FcRn^{f}_{endo-2} \times C^{f}_{endo-2} \times V_{endo-2} + K_{off(6.0)} \times C^{b}_{endo-2} \times V_{endo-2} - \frac{1}{\tau} \times C^{f}_{endo-2} \times V_{endo-2} - K_{deg} \times C^{f}_{endo-2} \times V_{endo-2}$$

Equation S8. Bound antibody in the second endosomal subspace

$$\textbf{Bound mAb: } V_{endo-2}\frac{dC^{b}_{endo-2}}{dt} = \frac{1}{\tau} \times C^{b}_{endo-1} \times V_{endo-1} + K_{on(6.0)} \times FcRn^{f}_{endo-2} \times C^{f}_{endo-2} \times V_{endo-2} - K_{off(6.0)} \times C^{b}_{endo-2} \times V_{endo-2} - \frac{1}{\tau} \times C^{b}_{endo-2} \times V_{endo-2}$$

Equation S9. Free FcRn in the second endosomal subspace

$$\textbf{Free FcRn: } V_{endo-2}\frac{dFcRn^{f}_{endo-2}}{dt} = \frac{1}{\tau} \times FcRn^{f}_{endo-1} \times V_{endo-1} - K_{on(7.4)} \times FcRn^{f}_{endo-2} \times C^{f}_{endo-2} \times V_{endo-2} + K_{off(7.4)} \times C^{B}_{endo-2} \times V_{endo-2} - \frac{1}{\tau} \times FcRn^{f}_{endo-2} \times V_{endo-2}$$

Equation S10. Free antibody in the third endosomal subspace

$$\textbf{Free mAb: } V_{endo-3}\frac{dC^{f}_{endo-3}}{dt} = \frac{1}{\tau} \times C^{f}_{endo-2} \times V_{endo-2} - K_{on(7.4)} \times FcRn^{f}_{endo-3} \times C^{f}_{endo-3} \times V_{endo-3} + K_{off(7.4)} \times C^{b}_{endo-3} \times V_{endo-3} - R2 \times C^{f}_{endo-3} \times V_{endo}$$

Equation S11. Bound antibody in the third endosomal subspace

$$\textbf{Bound mAb: } V_{endo-3}\frac{dC^{b}_{endo-3}}{dt} = \frac{1}{\tau} \times C^{b}_{endo-2} \times V_{endo-2} + K_{on(7.4)} \times FcRn^{f}_{endo-3} \times C^{f}_{endo-3} \times V_{endo-3} - K_{off(7.4)} \times C^{b}_{endo-3} \times V_{endo-3} - R2 \times C^{B}_{endo-3} \times V_{endo}$$

Equation S12. Free FcRn in the third endosomal subspace

$$\textbf{Free FcRn: } V_{endo-3}\frac{dFcRn^{f}_{endo-3}}{dt} = \frac{1}{\tau} \times FcRn^{f}_{endo-2} \times V_{endo-2} - K_{on(7.4)} \times FcRn^{f}_{endo-3} \times C^{f}_{endo-3} \times V_{endo-3} + K_{off(7.4)} \times C^{B}_{endo-3} \times V_{endo-3} - R2 \times FcRn^{f}_{endo-3} \times V_{endo}$$

Equation S13. Antigen concentration at steady state

$$K_{syn,antigen} = K_{deg,antigen} \times C_{antigen,t=0}$$

Equation S14. Antigen-antibody association rate, dissociation rate, and internalization rate of the complex in the interstitial space

$$Association\text{-}Rate = K_{on,antigen} \times (C_{antigen} - C_i^b) \times C_i^f \times V_i$$

$$Dissociation\text{-}Rate = K_{off,antigen} \times C_i^b \times V_i$$

$$Complex\text{-}Internalization\text{-}Rate = K_{int,TMD} \times C_i^b \times V_i$$

Equation S15. Change in antigen concentration due to binding and antigen-antibody complex internalization

$$V_i \frac{dC_{antigen}}{dt} = K_{syn,antigen} \times V_i - K_{deg,antigen} \times (C_{antigen} - C_i^b) \times V_i - K_{int,TMD} \times C_i^b \times V_i$$

Section S2. Sensitivity Analysis on TNF-α Levels in the Infliximab PBPK Model

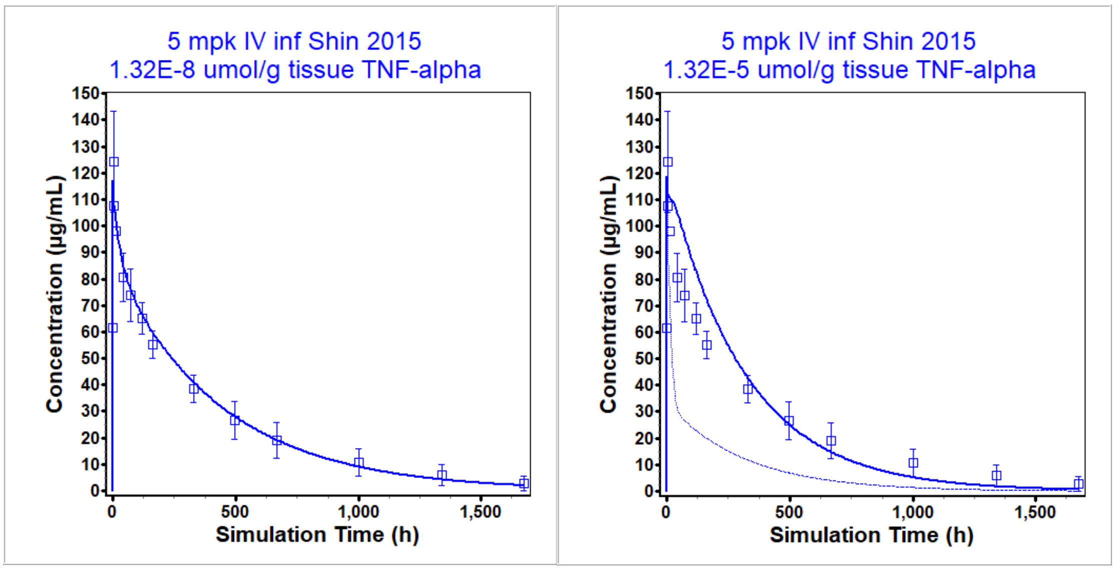

(**a**) (**b**)

Figure S1. Increasing TNF-α levels from the measured 5 pg/ml in plasma (1.32E-10 µmol/g tissue in blood) in healthy individuals to 500 pg/ml in plasma (1.32E-8 µmol/g tissue in blood) (a) showed no difference in predicted total and unbound infliximab concentrations (model prediction lines lie on top of each other). This value is well above 30 pg/ml which is the measured TNF-α concentration in plasma from RA patients [21]. Increasing TNF-α concentrations several orders of magnitude above 500 pg/ml to 500,000 pg/ml in plasma (1.32E-5 µmol/g tissue in blood) (b) does produce a difference between predicted total (dark blue) and unbound (light blue) infliximab concentrations but is outside of a physiologically relevant range.

Section S3. Sensitivity Analysis on PD-1 Levels in the Nivolumab PBPK Model

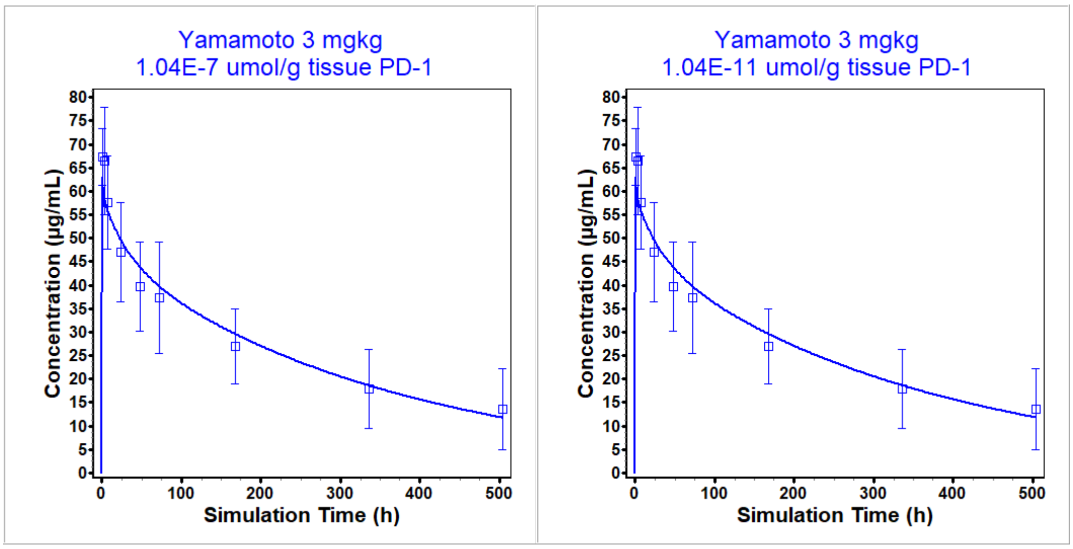

(**a**) (**b**)

Figure S2. A sensitivity analysis of two orders of magnitude above to 1.04E-7 µmol/g tissue of PD-1 in blood (a) and below to 1.04E-11 µmol/g tissue of PD-1 in blood (b) the average PD-1 concentration measured in cancer patients of 1.035E-9 µmol/g tissue [39] found no difference in predicted total and unbound nivolumab concentrations. The measured values of PD-1 in cancer patients were well within two orders of magnitude of the average [39].
